# Large-scale untargeted LC-MS metabolomics data correction using between-batch feature alignment and cluster-based within-batch signal intensity drift correction

**DOI:** 10.1007/s11306-016-1124-4

**Published:** 2016-09-22

**Authors:** Carl Brunius, Lin Shi, Rikard Landberg

**Affiliations:** 1 Department of Food Science, Uppsala BioCenter, Swedish University of Agricultural Sciences, Box 7051, 750 07 Uppsala, Sweden; 2 Unit of Nutritional Epidemiology, Institute of Environmental Medicine, Karolinska Insitutet, Box 210, 171 77 Stockholm, Sweden; 3 Department of Biology and Biological Engineering, Chalmers University of Technology, 412 96 Göteborg, Sweden

**Keywords:** Metabolomics, LC-MS, Data correction, Batch alignment, Drift correction

## Abstract

**Introduction:**

Liquid chromatography-mass spectrometry (LC-MS) is a commonly used technique in untargeted metabolomics owing to broad coverage of metabolites, high sensitivity and simple sample preparation. However, data generated from multiple batches are affected by measurement errors inherent to alterations in signal intensity, drift in mass accuracy and retention times between samples both within and between batches. These measurement errors reduce repeatability and reproducibility and may thus decrease the power to detect biological responses and obscure interpretation.

**Objective:**

Our aim was to develop procedures to address and correct for within- and between-batch variability in processing multiple-batch untargeted LC-MS metabolomics data to increase their quality.

**Methods:**

Algorithms were developed for: (i) alignment and merging of features that are systematically misaligned between batches, through aggregating feature presence/missingness on batch level and combining similar features orthogonally present between batches; and (ii) within-batch drift correction using a cluster-based approach that allows multiple drift patterns within batch. Furthermore, a heuristic criterion was developed for the feature-wise choice of reference-based or population-based between-batch normalisation.

**Results:**

In authentic data, between-batch alignment resulted in picking 15 % more features and deconvoluting 15 % of features previously erroneously aligned. Within-batch correction provided a decrease in median quality control feature coefficient of variation from 20.5 to 15.1 %. Algorithms are open source and available as an R package (‘batchCorr’).

**Conclusions:**

The developed procedures provide unbiased measures of improved data quality, with implications for improved data analysis. Although developed for LC-MS based metabolomics, these methods are generic and can be applied to other data suffering from similar limitations.

**Electronic supplementary material:**

The online version of this article (doi:10.1007/s11306-016-1124-4) contains supplementary material, which is available to authorized users.

## Introduction

Untargeted metabolomics aims to profile the global metabolome, i.e. the (semi-)quantitative collection of low molecular weight metabolites within a biological system, usually in biofluids such as urine, serum, plasma or tissue/cellular extracts (Shulaev 2006; Patti et al. 2012; Vinayavekhin and Saghatelian 2010; Yin and Xu 2014; Alonso et al. 2015). Metabolomics thus finds its place downstream of genomics and proteomics and represents the omics technique closest to phenotype, through the interactions of the previous omics levels with the exposome (Scalbert et al. 2014; Rappaport et al. 2014). Over the past decade, it has become an increasingly used tool in biological and medical research through possibilities offered for predictive biomarker discovery, elucidation of metabolic pathway alterations and disease aetiology and reflection of demography, lifestyle and exposures (Dunn et al. 2011; Matsuda et al. 2009; Bajad and Shulaev 2011; Beckmann et al. 2013; Dunn et al. 2012; Rappaport et al. 2014). Among the different techniques employed in metabolomics, untargeted liquid chromatography-mass spectrometry (LC-MS) is extensively used due to its high sensitivity, simple sample preparation and broad coverage of metabolites (Theodoridis et al. 2008; Fernandez-Albert et al. 2014; Bajad and Shulaev 2011). Until recently, mass spectrometric techniques were not sufficiently reproducible for large-scale untargeted metabolomics studies involving thousands of samples. However, advances in instrumentation, experimental protocols and data processing methods now permit the use of LC-MS in large-scale untargeted studies with thousands of samples for analysis (Ganna et al. 2014; Drogan et al. 2014).

Data from large-scale LC-MS based metabolomics experiments are generally collected over long periods and analysed in multiple batches. The data collected are affected by systematic and random variability in signal sensitivity, mass accuracy (*m/z*) and retention times (*rt*) between samples both within and between batches. This variability gives rise to critical challenges regarding information loss and data processing (Dunn et al. 2011, 2012).

Within- and between-batch variations in signal intensity (Warrack et al. 2009; Sysi-Aho et al. 2007; Dunn et al. 2011) contribute to noise in the data and therefore have a negative impact on statistical analysis and consequently on the discovery and accurate quantification of metabolites of interest (Veselkov et al. 2011; Wang et al. 2013). Shifts in *m/z* and *rt* of molecular features between analytical runs result in different extracted spectrum patterns for a single metabolite across samples, with potential misalignment as a consequence. Such drifts could therefore severely affect subsequent statistical analysis and further metabolite identification (America et al. 2006; Nordström et al. 2006; Lange et al. 2008; Zhang et al. 2015). In a recent review (Smith et al. 2015), the state-of-the-art on peak alignment algorithms is well summarised. Unfortunately, current algorithms still suffer from shortcomings, especially regarding between-batch misalignment, since *m/z* and *rt* shifts are generally much larger between batches than within. Moreover, to the best of our knowledge there are no available methods to specifically address systematic misalignment across multiple batches. Improved algorithms are thus urgently needed.

Different approaches for signal intensity drift management are available. A common approach is to include internal standards (Bijlsma et al. 2006; Sysi-Aho et al. 2007), but this may not be feasible for untargeted metabolomics studies since available internal standards only represent a limited number of metabolites and signal intensity fluctuations may differ between various metabolite classes (Ejigu et al. 2013; Dunn et al. 2012; Vinayavekhin and Saghatelian 2010). In large-scale untargeted metabolomics studies, the most simple normalisation methods are based on total intensity or intensity of the most stable features. However, these methods are questionable since they assume similar intensity shifts for all features between samples and consequently perform less well than feature-based normalisation techniques (Kamleh et al. 2012). Slightly more advanced methods include e.g. quantile normalisation techniques, which are based on the assumption of similarity of signal intensity distributions, rather than the intensities themselves (Kohl et al. 2012; Lee et al. 2012). However, these methods do not take into account specific feature drift patterns or different signal intensity distributions between different sample classes (e.g. case-control). More recently, quality control (QC) sample strategies have been commonly applied in signal drift management (Kirwan et al. 2013; Dunn et al. 2011, 2012; Kamleh et al. 2012). QC samples have a matrix composition similar to that of the biological samples to be studied, normally achieved by pooling aliquots of the study samples. These QC samples are then injected randomly or regularly within batches to evaluate the LC-MS system and data pretreatment performance, followed by algorithms aiming to discard noisy features or to reduce sample-to-sample or batch-to-batch variations in signal intensity (Dunn 2012; Nezami Ranjbar et al. 2013; Fernandez-Albert et al. 2014; Dunn et al. 2011; Kamleh et al. 2012; Kirwan et al. 2013).

In the present work we introduce two new approaches for overcoming the above-mentioned obstacles regarding processing multiple batch LC-MS metabolomics data, i.e. between-batch feature alignment and within-batch cluster-based drift correction. We also introduce a heuristic suitability criterion to aid in the choice of reference-based or population-based between-batch signal intensity normalisation per feature. Although these approaches are designed for untargeted LC-MS metabolomics, they can be extended to other areas of chemical analysis, such as GC-MS or LC-MS, and for purposes other than metabolomics where signal intensity drift and alignment issues may occur.

## Materials and methods

Throughout this article, the term ‘feature’ refers to a mass spectral peak, i.e. a molecular entity with a unique m/z and retention time as measured by an LC-MS instrument, such as a metabolite ion, isotope, adduct, fragment or random noise.

### Data set

Fasting plasma samples with heparin as anticoagulant originating from a type 2 diabetes (T2D) case-control study nested within the Northern Sweden Health and Disease Study Cohort (Norberg et al. 2010) were obtained from the Medical Biobank in Umeå (Hallmans et al. 2003). The study was approved by the regional ethical review board in Uppsala (Dnr 2011/228). The samples (n = 503) were drawn in 1991–2005 from men and women who later developed T2D, for whom previously unthawed plasma samples were available, and from individually matched controls who remained free of diabetes until the end of follow-up. Additional fasting plasma samples (n = 187) taken 10 years later from controls were used to assess the long-term stability of potential biomarkers and repeat samples from cases (n = 187) were analysed to assess potential changes in metabolites related to the risk of developing of T2D. Instrument analyses were performed with approximately 250 samples per batch (including QC and reference samples) in eight batches over 6 months. In the instrumental analysis protocol, two independent biological sample types were used to monitor the stability and functionality of the system throughout all analyses. These were: batch-specific quality control samples (QCs), i.e. pooled plasma samples of all biological samples within batch; and long-term reference samples, i.e. pooled plasma samples of healthy people stored and offered by the Institute of Public Health and Clinical Nutrition, Kuopio, Finland, consistently used throughout all batches. The reference samples were thus not of the same biological origin as the samples and QCs and, unlike the QCs, were therefore not directly representative of the sample population. The QCs and reference samples were injected at the beginning and end and as every 14th injection throughout each batch sequence, and together constituted approximately 16 % of analytical samples. The LC-MS data used were taken from three of the eight batches, selected randomly, and constituted a subgroup of quality monitoring samples, including 48 QCs and 42 reference samples.

LC-MS based metabolomics was conducted in collaboration with the metabolomics platform at the University of Eastern Finland. Preparation of fasting plasma samples for metabolite profiling followed the procedure described by Hanhineva et al. (2015b). In brief, 90 μL sample was mixed with 360 μL acetonitrile, incubated in an ice bath for 15 min and then centrifuged at 1200 g through 0.2 μm polytetrafluoroethylene filters. After 5 min, clear, de-proteinated filtrate was collected for analysis. Plasma samples were analysed by ultra-high performance liquid chromatography quadrupole time-of-flight mass spectrometry (UHPLC-qTOF-MS, Agilent Technologies). The system consisted of a 1290 LC system, a Jetstream electrospray ionisation source and a 6540UHD accurate mass qTOF spectrometer operating in positive ionisation mode. The procedure for sample analysis was as described in detail by Hanhineva et al. (2015b), with modification. In brief, 4 μL of the sample solution were injected on the column (Zorbax Eclipse XDB-C18, 2.1 × 100 mm, 1.8 µm) operating at 50 °C. The mobile phase was delivered in a reversed-phase gradient elution at 0.4 mL/min, using water (eluent A) and methanol (eluent B), both containing 0.1 % formic acid. The following gradient profile was used: 2/100 % B (0–10 min), 100 % B (10–14.5 min), 100/2 % B (14.5 min), 2 % B (14.5–16.5 min). The MS conditions were set up as previously described (Hanhineva et al. 2015b) and the instrument scanned from 20 to 1600 m/z. Data were collected in centroid mode at an acquisition rate of 1.67 spectra/s with an abundance threshold of 150.

Instrument data were exported to ‘xml’ file format and processed in the R open source environment (v 3.2.0; R core team 2016) using the XCMS package (Smith et al. 2006; Tautenhahn et al. 2008). XCMS peak picking parameters (prefilter, peakwidth, mzdiff, snthresh) were obtained using the IPO R package (Libiseller et al. 2015). Final peak picking parameters were: prefilter = c(3,440), peakwidth = c(5,76), snthresh = 6, mzdiff = 0.0045, ppm = 15. Initial alignment (bw = 15, minfrac = 0.75, minsamp = 1, mzwid = 0.015) and retention time correction (standard loess, family = “s”, span = 0.2) were then applied. For the final alignment, bw was set to the largest observed retention time deviation from visual inspection of XCMS retention time correction plots obtained within batch. Consequently, final alignment was applied with parameters: bw = 1, mzwid = 0.015, minfrac = 0.75, and was followed by filling in missing peaks (method = ‘chrom’).

### Feature alignment between batches

**Fig. 1:**
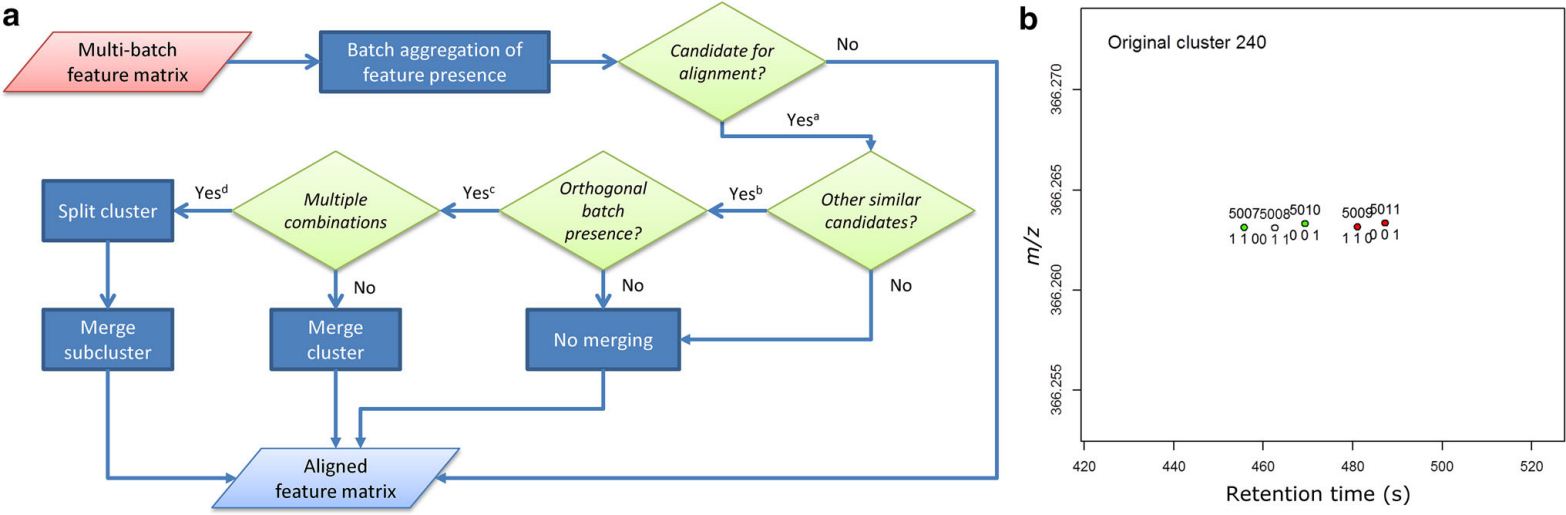
Proposed algorithm for between-batch feature alignment. **a** Flowchart for alignment of features systematically misaligned between batches. ^a^A feature is considered a potential candidate for alignment if 0 < total batch presence < number of batches. ^b^Alignment candidates are considered similar if having *m/z* and *rt* within user-defined tolerance. ^c^Candidates are considered for alignment and subsequently clustered if not mutually present in the same batch (i.e. presence vector orthogonality). ^d^Clusters containing multiple possible alignments are recursively subdivided into sub-clusters. **b** Deconvolution of multiple batch alignment candidates within a selected cluster. During peak picking, five molecular features (numbers 5007–5011) were detected within the specified *m/z* and *rt* tolerance, in samples from three analytical batches. Feature presence/absence was aggregated on batch level and marked as absent if missing from >80 % of QC samples per batch. Presence (1) or absence (0) is noted in the figure for the five features in vector format, where each position in the vector corresponds to presence/absence in the three batches. Candidates for alignment (i.e. the *four colour-filled* features) were identified through (i) proximity in the *m/z* and *rt* domains (i.e. closeness) and (ii) orthogonality of presence vectors (i.e. two alignment candidates cannot be present in the same batch). Note the *unfilled* feature number 5008, which due to the second criterion is excluded as a possible alignment candidate. Multiple alignment candidates were sub-clustered (*different colour-filled* features) through a recursive deconvolution algorithm

Alignment of features systematically misaligned between batches was performed in a multistep algorithm (Fig. 1a). First, to investigate systematic missingness and filter out random noise inherent in individual samples, feature missingness was aggregated on batch level. This was done by batch-wise per-feature calculation of the proportion of missingness among the reference samples and flagging batch absence for those features satisfying the criterion (Eq. 1):

1 $$proportion_{NA, feature, batch} = \frac{{nSamples_{NA, feature, batch} }}{{nSamples_{Total, batch} }} > 80\%$$

where *NA* denotes missing value and *nSamples* denotes the number of samples within a batch. The 80 % limit was chosen as an extension of the 80 % rule often employed in metabolomics (Smilde et al. 2005). Batch presence was similarly flagged as non-absence. Candidates for batch alignment were those features where the sum of presence flags exceeded zero (representing being missing from all batches, thus with no possibility for alignment) and lower than the total number of batches (representing being present in all batches, thus with no possibility for alignment).

For each candidate, correspondence with other candidates (“events”) was investigated through distance, i.e. within a user-defined box bounded by largest allowed absolute *m/z* and *rt* differences under the constraint of batch presence being orthogonal between features, i.e. ensuring that two features present in the same batch cannot be aligned. The boundary value for *m/z* (0.002 Da) was set according to instrument resolution and *rt* (15 s) was determined from maximum retention drift between batches obtained from XCMS.

All distinct events thus consisted of two alignment candidates. Events which shared common alignment candidates were then clustered. In the case where all such cluster candidates were mutually orthogonal, correspondence was assumed and alignment candidates would then be merged. However, in some of these clusters multiple alignment combinations were possible (Fig. 1b; All coloured features), indicating correspondence to more than one underlying feature. Multiple alignment candidates were in that case disentangled into their respective correspondences through a recursive sub-clustering algorithm: The largest distances per cluster were iteratively removed until single possible alignment candidates (sub-cluster, i.e. unique correspondence) could be identified. All other possible alignment candidates per cluster, including those previously removed, then underwent the same recursive algorithm until no further sub-clustering could be achieved (Fig. 1b; Different colours for different sub-clusters, i.e. unique correspondences).

### Cluster-based within-batch drift correction

**Fig. 2:**
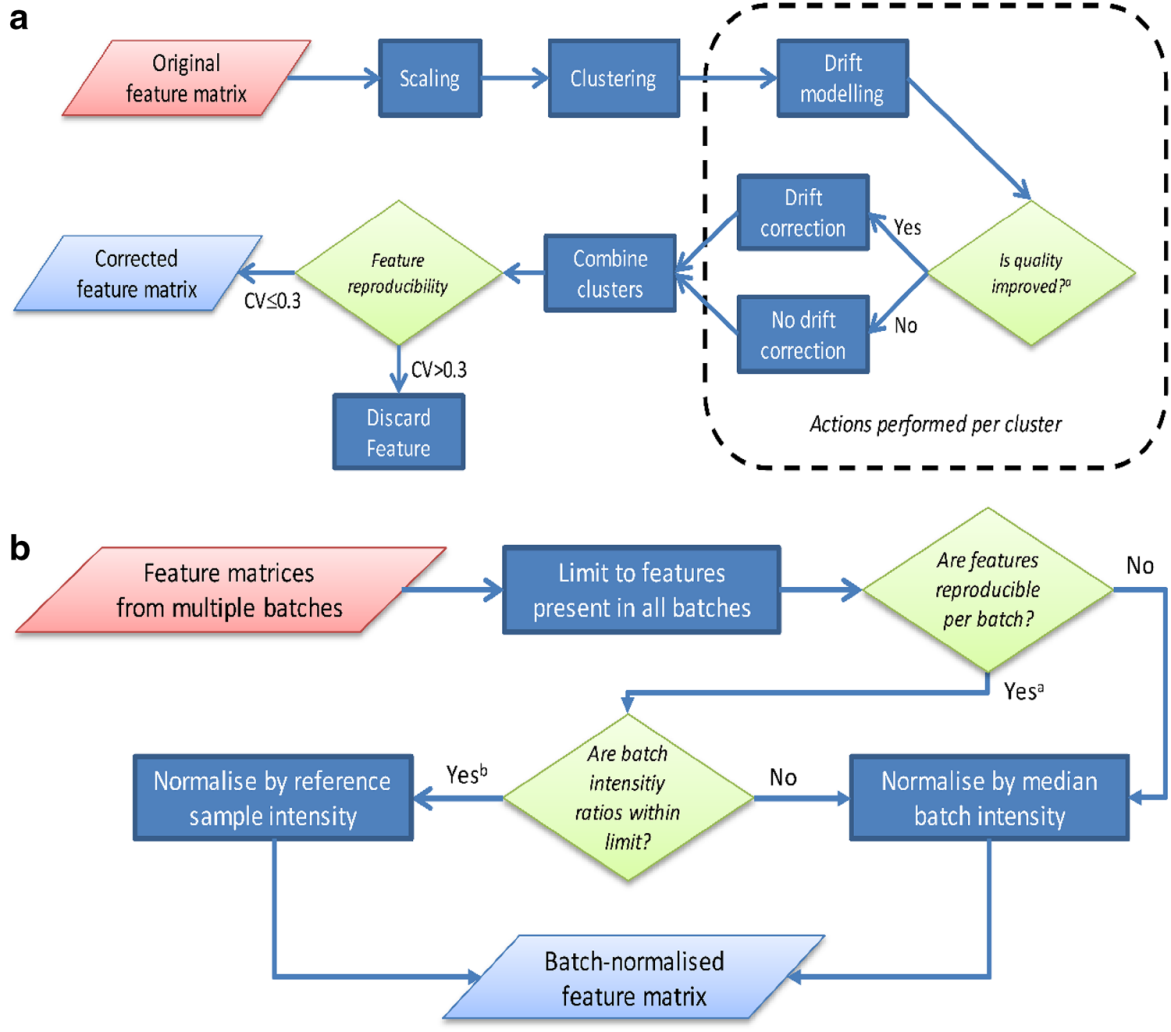
Proposed algorithms for within- and between-batch signal intensity drift corrections. **a** Flowchart of cluster-based within-batch intensity drift correction. ^a^In this example, cluster quality is considered to be improved if rmsd(*Ref*)_with correction_ < rmsd(*Ref*)_without correction_, where ‘rmsd’ denotes root mean squared distance from the cluster centre point. *Ref* denotes long-term reference samples not used for within-batch intensity drift modelling (see Materials and methods section). **b** Flowchart of Between-batch intensity normalisation algorithm. ^a^Features are considered reproducible if long-term reference sample intensity per batch CV ≤ 30 %. ^b^Reference sample average feature intensity ratios between batches are considered within limit if not deviating from corresponding average feature intensity ratios by more than a fold change of five. For such features passing both criteria, batches are normalised by average reference sample intensity. For other features, long-term reference samples are not considered sufficiently representative of the sample population and features are thus normalised by median batch intensity

The multi-batch data were separated into batch-specific subsets and within-batch drift correction was performed separately on each of these subsets in an algorithm consisting of four distinct steps (Fig. 2a): Clustering of features; drift modelling per cluster; drift correction per cluster; and removal of individual features with poor reproducibility after cluster-based drift correction.

To facilitate clustering of variables, data were first scaled by standard deviation but not centred, under the assumption of predominantly multiplicative rather than additive error terms in instrumental chemical analysis. Clustering of variables was performed under the assumption that variables with similar drift pattern, in addition to being strongly correlated, are characterised by small Euclidean distances when seen as coordinates in the multivariate sample (or observation) space as opposed to viewing samples as observations in the variable space, which is normally performed in multivariate statistical modelling. A visualisation of distinct drift patterns using lower-dimensionality synthetic data is available as supplementary material (Suppl. Table 1, Figs. 1 and 2).

Clustering of variables in the observation space was achieved by employing the “mclust” algorithm, which utilises a Bayesian approach to determine the type (i.e. geometrical constraint) and optimal number of clusters inherent in the data (Fraley and Raftery 2002; Fraley et al. 2012). This algorithm was chosen to decrease operator bias in the clustering operation. First, a wide range of geometrical constraints in clusters are available, together with the ability to specify a range in potential numbers of cluster to examine. In the mclust algorithm, final cluster parameters are automatically chosen from the Bayesian Information Criterion (BIC) values from all combinations of user-supplied parameter values (cluster type and number) and a BIC plot is optionally produced for a visual overview of clustering performance for the available parameter combinations. It should be noted, however, that for large, multidimensional data, mclust is a computationally expensive algorithm. Initial testing revealed that restricting the model type to ‘VVE’, *i.e*. ellipsoidal clusters with equal orientation and number of clusters from 1 to 52 in steps of three, provided a good balance between high quality, unbiased clustering and computational cost. Applying these parameter settings, clustering of 12–18 QC samples × 11 815 features resulted in 25–28 clusters and required approximately 12–18 min in the present case.

After clustering, scaled variables belonging to the same cluster were pooled together and a cubic spline regression was applied on the pooled cluster data vs injection order to obtain a drift function. The algorithm performs this drift calculation separately for all the clusters and optionally produces plots of the clusters and their correction functions (Fig. 2a, Suppl. Fig. 3). Finally, cluster-based drift correction was achieved by calculation of cluster-wise correction factors (Eq. 2) applied specifically for each injection:

2 $$correctionFactor_{c,n} = \frac{{driftValue_{c,1} }}{{driftValue_{c,n} }}$$

where *correctionFactor* are the cluster-based correction factors derived from the corresponding drift function values *driftValue* for cluster *c* at injection *n*.

These correction factors are derived from the ratio between drift function values at the reference point (i.e. first injection) and at all subsequent injections obtained from the cluster drift function. By multiplying these correction factors to the original unscaled variable data of the cluster, intensity drift was thus normalised to the reference level at the first injection (Suppl. Fig. 3).

Drift correction per cluster was performed only if providing an unbiased measure of increased quality of data measured on non-QC reference samples. This was assessed by cluster-wise evaluation of the root-mean-squared distance (rmsd) from the centre point of the long-term reference samples with and without correction. This provided an unbiased measure since: (i) the long-term reference samples were of different biological origin than the QC samples and (ii) they were not included in drift modelling and correction. Correction per cluster was thus only performed if the rmsd was reduced after drift correction. After drift correction, individual features were removed batch-wise from the subset if QC sample CV_After correction_ > 30 %.

### Between-batch normalisation

Between-batch normalisation was performed through an iterative process (Fig. 2b). Aggregated batch data after within-batch drift correction and alignment were first limited to common features, i.e. those features common between all batches that were not excluded after cluster-based drift correction per batch. Normalisation was then achieved using either of two standard approaches: Normalisation by reference sample intensity or population-based (median) normalisation. However, to aid in the feature-wise choice between normalisation methods, a heuristic was developed and applied to test for suitability of batch normalisation by reference sample intensity. Normalisation by average reference sample intensity per batch and feature was performed if satisfying the following dual criterion:

3 $$CV_{Ref,\,batch } < 0.3 \left\{ {for\, all \,batches} \right\}$$

4 $$log\left( {\frac{{Feature\, Intensity\, Ratio_{i,j} }}{{Average\, Feature\, Intesity\, Ratio_{i,j} }}} \right) < \log 5 \left\{ {for\, all\, batches\, i,j} \right\}$$

where *Feature Intensity Ratio*_*i,j*_ is the ratio of average reference sample intensity for a specific feature measured in batches *i* and *j*, respectively and *Average Feature Intensity Ratio*_*i,j*_ is the ratio for the average intensity of all features within the batches *i* and *j*, respectively. The use of log-transformation on each side of Eq. 4 is to provide equidistant fold changes for batches *i* and *j*. A fold change limit of 5 was used for this data, but the limit can be user-defined (see Results and discussion). When the criterion was not met, the reference samples were not considered sufficiently representative of the sample population or otherwise inadequate for normalisation, in which case batches were normalised by median intensity of batch sample populations under the assumption of similar population distributions between batches (data not shown).

### Computer hardware and software

Algorithms were developed in the open source statistical software environment R v 3.2.2 (R Core Team 2016) and depended on the following non-base packages: “mclust” v 5.0.1 (Fraley and Raftery 2002; Fraley et al. 2012), “reshape” v 0.8.5 (Wickham 2007) and “XCMS” v 1.44.0 (Smith et al. 2006; Tautenhahn et al. 2008). All calculations were performed on a HP Elitebook with an Intel i7-3687U processor operating under Windows 7. Functions and data sets are available as an R package (*batchCorr*) and, together with example data and script, are freely available from https://gitlab.com/CarlBrunius/batchCorr.

## Results and discussion

Multi-batch high-resolution LC-MS data present challenges in terms of signal intensity drift and feature misalignment from instrument deviations in the *m/z* and *rt* domains. These deviations have contributions both from within- and between-batch irregularities, with the latter generally expected to make a greater contribution. Data sets should consequently be first examined for systematic feature misalignment between batches to avoid loss of data integrity, the type of which depends on the data pipeline employed. In the case of forced integration data filling (such as the *fillPeaks* algorithm in the XCMS package) and data imputation, there is the obvious risk of splitting one true informative feature into two or more with either less or wrong information (Suppl. Fig. 4). Data filling creates artificially batch-specific features from the systematic differences in presence/missingness, i.e. erroneous information, whereas imputation in the best of cases results in two (or more) identical features, although most imputation techniques would struggle to achieve accurate imputations with this type of batch-specific systematic missingness, thus increasing noise in the variables and consequently in following statistical analyses, i.e. providing/introducing less and/or wrong information.

Available options for feature alignment (Smith et al. 2015) do not distinguish within-batch from between-batch deviations and are therefore best suited for within-batch alignment of features. To investigate and correct for systematic batch structures, presence/missingness was aggregated per batch to filter out spurious random noise or erroneous misalignment of individual samples. Batch absence was decided employing an 80 % threshold (Bijlsma et al. 2006), but robust results were in fact obtained between threshold settings of 60–85 % (data not shown).

To be considered for alignment, features should be sufficiently close in the *m/z* and the *rt* domains and also have orthogonal feature vectors. Using features from Fig. 1b as an example: Feature 5007, flagged as [1 1 0] would be present in batches 1 and 2, but not in batch 3, whereas feature 5010, flagged as [0 0 1] would be orthogonally present and therefore a possible alignment candidate. In the majority of cases, alignment candidates were only involved with one other candidate. However, multiple alignment combinations within the same *m/z–rt* box were also observed (Fig. 1b; All coloured features), possibly due to extreme similarity in retention time of e.g. stereoisomers. Several approaches for disentanglement of such multiple alignment candidates are possible. The naïve option to choose the alignment candidates with the shortest distance in the *m/z–rt* box resulted in apparent misalignments (data not shown). A recursive sub-clustering algorithm was therefore developed to identify all unique correspondences (Fig. 1b; different colours per correspondence). The algorithm also optionally produces plots of alignment events and sub-clusters. The effectiveness of the clustering and sub-clustering algorithms was confirmed by visual inspection of clustering and sub-clustering results.

This algorithm for systematic batch misalignment is easily integrated with available sample-based alignment methods. Within an analytical batch, *m/z* and *rt* can be expected to remain within small tolerances relative to between-batch variation, especially in the *rt* dimension. For metabolomics data spanning several batches, low tolerance settings thus increase the risk of misalignment, whereas high tolerance risks binning unrelated features. Peak alignment in workflows not addressing batch alignment thus necessitates increasingly higher tolerance settings with the number of batches being combined. In a workflow employing batch-orthogonal alignment, between-sample alignment can instead be optimised per batch, thus with narrower *m/z* and *rt* setting, and later combined using batch-aggregated data (Suppl. Fig. 4). For comparison, using the current dataset and a bw = 15 setting, this corresponded to picking approximately 9800 features (Suppl. Fig. 5). Using settings optimised for batch-specific peak picking (bw = 1) and systematic batch alignment, approximately 11,300 were instead picked. The advantage was two-fold: (i) 1500 features previously aligned were deconvoluted, contributing to noise reduction in 1500 of the previously available features; and (ii) 1500 additional features were added to the dataset, increasing the information content. It should be noted that by decreasing bandwidth setting alone, there is no effective means to distinguish between true feature deconvolution and artificially splitting features between batches. In fact, without the batch alignment algorithm, a bw = 1 setting picked approximately 11,800 features, of which approximately 500 thus resulted from artificially splitting true features between batches (Suppl. Fig. 5).

For the within-batch signal intensity drift correction algorithm, clustering was automated to provide an unbiased trade-off between drift modelling detail and power through unbiased decision on optimum number of clusters through Bayesian clustering (Fraley and Raftery 2002; Fraley et al. 2012). It was observed that for multivariate, authentic data, model restrictions were not required to achieve reproducible clustering. Restrictions were however imposed to reduce computing time, without any apparent loss of information content. It should be noted that clustering could become computationally more efficient by parallelising cluster model calculations, which remains a point to consider in future algorithm development. Moreover, to increase computational efficiency, other clustering methods could also be considered. However, care should be taken to provide automated, unbiased estimations of optimal number of clusters and distance/clustering functions (Rokach 2009). It should be noted that a major limitation of the clustering is its poor capability for managing missing values, since the clustering is based on Euclidean distances in the multivariate observation space. If values are lacking, then these distances are effectively incalculable. Care must therefore be taken to provide full data matrices, i.e. peak tables without any missing data. This can be achieved either by forced integration between consensus peak limits such as performed by the ‘fillPeaks’ function of the XCMS package, or by imputation.

**Fig. 3:**
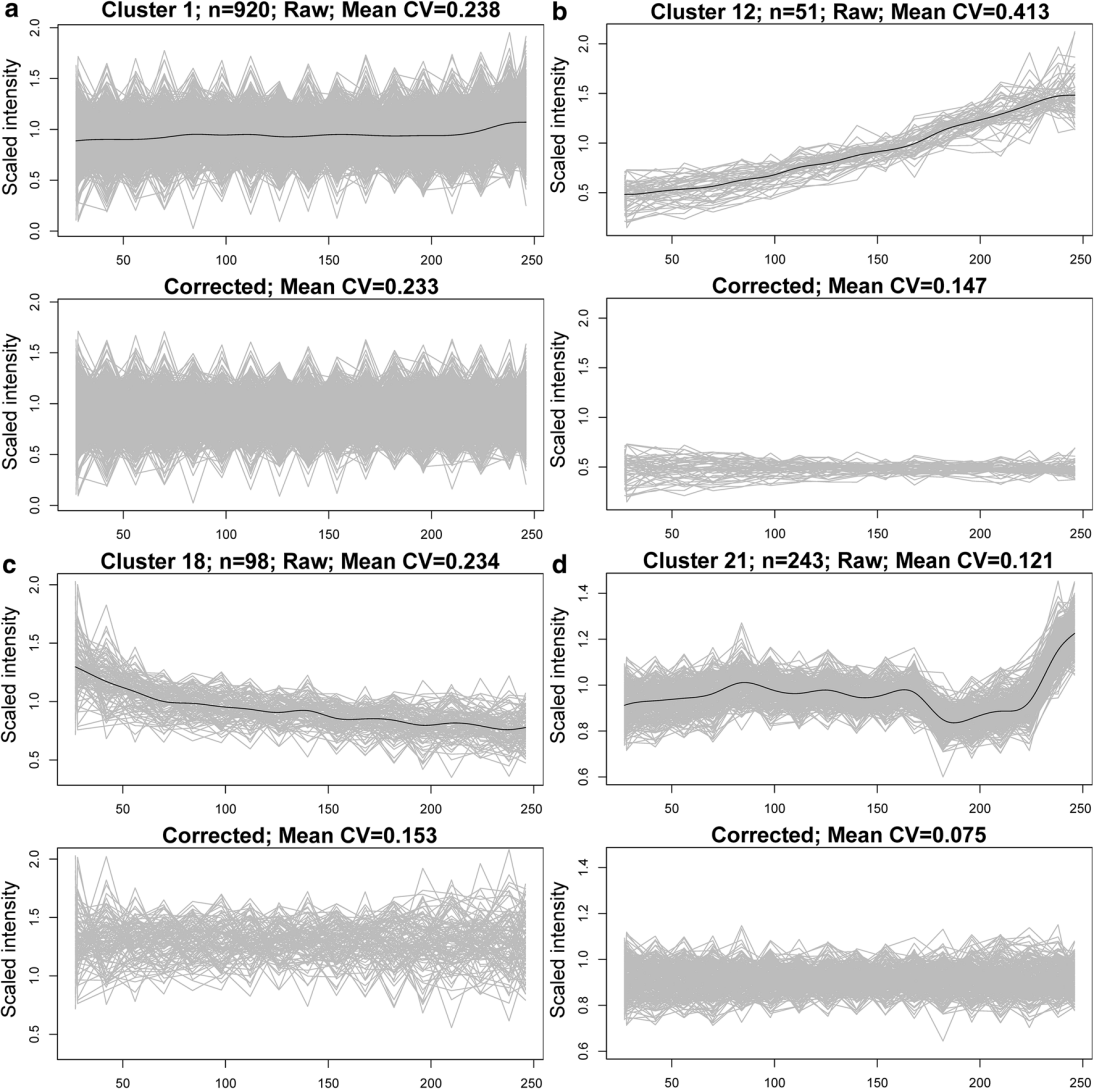
Authentic batch B QC features separated into different sized clusters (n = 51–920 features per cluster in this subset) representing different intensity drift behaviours. **a** Cluster 1, similarly to other large clusters (not shown), closely followed the general within-batch intensity drift, which for this batch was minor. Among the other clusters (**b**–**d**), several distinctly diverse drift patterns were readily discernible and CV was considerably reduced in those clusters. For each cluster, the upper graph shows the scaled features in *grey* and the cluster drift function in black. The lower half shows the same features on the same y-scale after application of cluster-based drift correction

Drift modelling was performed by cubic spline interpolation (Fig. 3). Smoothing functions are often sensitive to parameter settings, but due to the high number of data points per cluster compared with feature-wise interpolation, smoothing was highly reproducible for a wide range of parameter settings. Similar performance was observed using both local regression (LOESS) and cubic spline regression, but we found that cubic splines tended to be more insensitive to parameter fluctuations and thus less sensitive to operator bias.

**Fig. 4:**
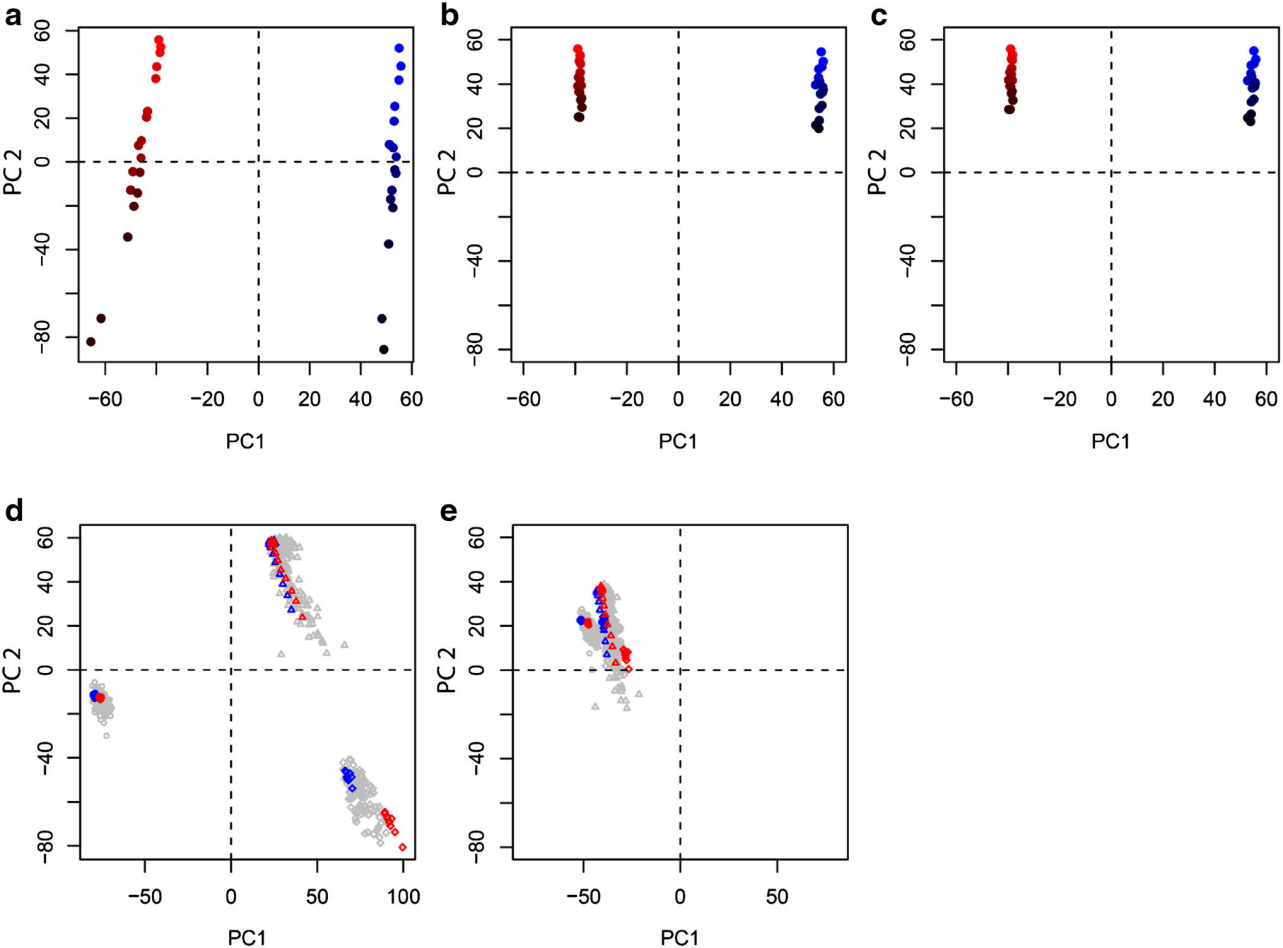
PCA score plots for performance of within-batch and between-batch drift correction. For within-batch drift correction (**a**–**c**) *red circles* represent within-batch QC samples and blue circles represent the long-term reference samples. *Color* is scaled corresponding to injection order. The drift observed in the raw, uncorrected data from batch B (**a**), is drastically reduced using cluster-based intensity drift correction either not employing (**b**) or employing (**c**) the fitness criterion of improved reference sample homogeneity and projected on the same scale as the uncorrected data. For between-batch drift correction (**d**–**e**), batches B,F and H are presented as *circles*, *triangles* and *squares*, respectively. *Red* represents within-batch QC samples, blue the long-term reference samples and *grey* the actual biological samples. The batch effect, clearly observed as the main determinant of variance prior to normalisation (**d**) is drastically reduced using the mixed normalisation procedure (**e**) when projected on the same scale

When observing the quality control samples in a principal component analysis (PCA), the intensity drift easily observed in the raw data from batch B (Fig. 4a) was drastically reduced when applying the cluster-based drift functions for signal correction (Fig. 4b). However, cluster-wise drift correction was performed only if it increased the quality of the data and such fitness estimation needs to rely on examination of quality monitoring samples not included in the modelling. In the present case, quality improvement was assessed as decreased Euclidean distance in the multivariate feature space between long-term reference samples (Fig. 4c).

Cluster 18 (Fig. 3) constituted an interesting example of this principle: Calculation of within-cluster drift provided a decrease in QC CV from 23 to 15 %. However, since the drift correction did not result in an increase in unbiasedly assessed quality of independent reference samples, the drift correction was not applied (Table 1). It should also be noted that the algorithm can easily be adapted to suit other schemes for fitness estimations and/or different experimental protocols, such as the use of duplicate (or multiple) injections of multiple samples or random subsampling among QC samples. Using multiple samples, fitness can e.g. be assessed through Euclidean distances with one-tailed paired tests of H1 < H0 (populations of distances before/after correction). In the latter case, QCs would be subsampled into two groups, for drift modelling and fitness estimation, respectively.

**Table 1:** Characteristics of clusters automatically identified by the cluster-based within-batch drift correction algorithm based on quality control (QC) samples from batch B (pooled human plasma samples; see Materials and methods for details) in reverse phase, positive electrospray ionisation UHPLC-QTOF

Cluster	Action^a^	Number of features		Mean QC feature CV (%)		
		Before^b^	After^c^	Before^b^	Correction^d^	After^c^
1	Corrected	920	920	24	23	23
2	Corrected	1177	1177	15	14	14
3	Corrected	715	7	36	35	29
4	Corrected	354	354	9	6	6
5	Corrected	860	860	13	11	11
6	Corrected	1137	1137	21	20	20
7	Corrected	771	771	10	9	9
8	Corrected	305	0	63	63	NA
9	No action	289	0	130	130	NA
10	Corrected	888	691	29	28	28
11	Corrected	309	309	26	25	25
12	Corrected	51	50	41	15	14
13	Corrected	198	198	21	12	12
14	Corrected	530	0	46	45	NA
15	Corrected	115	115	10	4	4
16	Corrected	154	154	30	16	16
17	Corrected	65	65	24	7	7
18	No action	98	83	23	15	21
19	Corrected	214	214	16	8	8
20	Corrected	107	107	12	8	8
21	Corrected	243	243	12	8	8
22	Corrected	1390	1390	18	17	17
23	Corrected	105	105	7	4	4
24	Corrected	221	221	8	6	6
25	Corrected	62	62	6	3	3

^a^Action performed on cluster: corrected if root-mean-squared distance (rmsd) of reference samples is decreased after applied correction, no action otherwise

^b^Values pertaining to original QC features

^c^Values pertaining to QC features after entire within-batch correction procedure (Fig. 5)

^d^CV of drift-corrected clusters

**Fig. 5:**
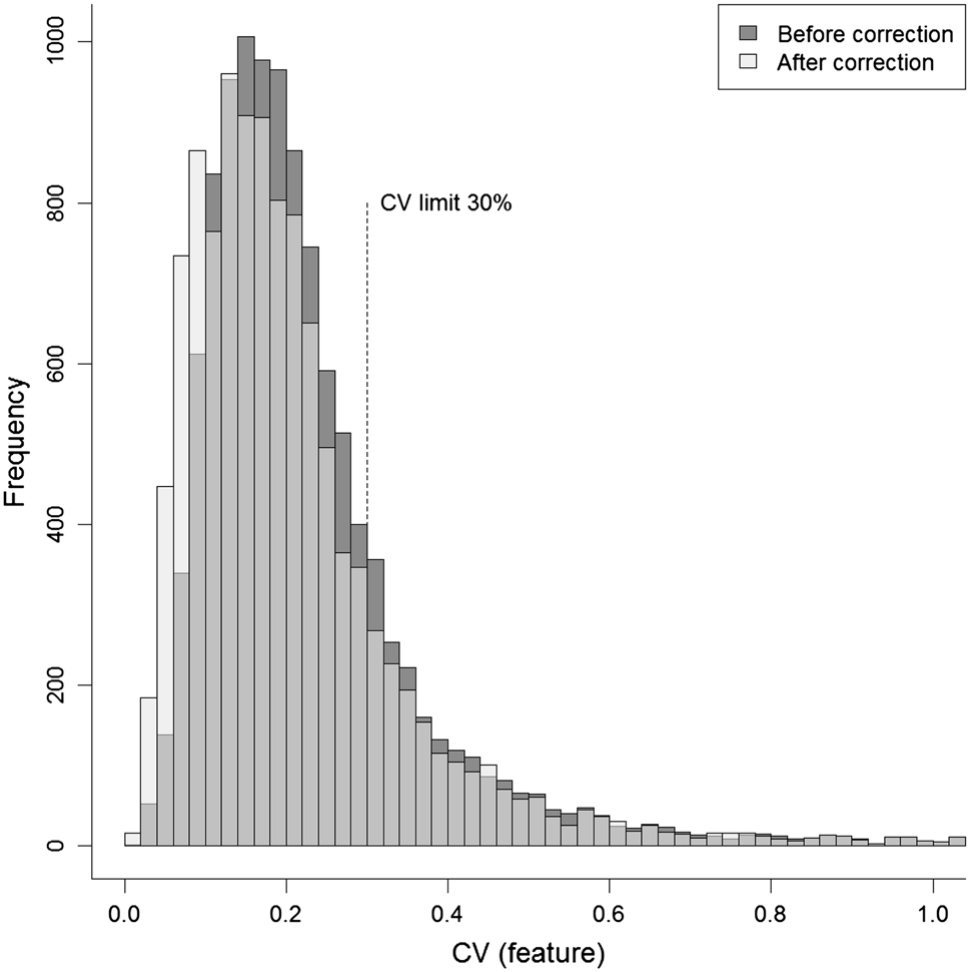
CV distribution among QC features before (*dark*) and after (*light*) cluster-based within-batch intensity drift correction of batch B of authentic LC-MS metabolomics data. Before correction, 8996 of originally 11,298 QC features (79.6 %) pass the CV ≤ 30 % criterion. After correction, the corresponding figure is 9233 features (81.7 %)

A final quality control of features was performed by removing individual features with CV > 30 % in QC samples after drift correction (Fig. 5). A notable effect of the clustering algorithm was the fact that features with poorer reproducibility, as indicated by final QC feature CV > 30 %, were in general clustered together (Table 1; clusters 3, 8, 9 and 14), as were features with common, highly reproducible drift patterns. It should also be noted that the correction algorithm may not be relevant for these clusters. However, in order to have a generalisable method, clustering is anyway applied if resulting in unbiasedly assessed increased data quality. According to the criteria, correction led to quality improvement in clusters 3, 8 and 14. However, the quality improvement was not so large as to warrant inclusion of most of these features in the final peak table. The final result is thus a combination of multiple sub-algorithms. In the three batches of the authentic data set, such clusters represented 13–19 % of the total number of features. However, some clusters (Table 1, clusters 3 and 14) consisted of both well and poorly behaving features.

Median QC feature CV for the three batches decreased from 20.5 to 18.7 % after drift correction and further to 15.1 % after feature removal. In many protocols, a CV limit of 20 % is applied, and also recommended in the FDA guidelines for bioanalytical method validation (FDA 2001). For the three batches in the authentic data set, a limit of 20 % resulted in 36–60 % of features being discarded. We advocate that a more accommodating setting of 30 % be applied in exploratory, untargeted metabolomics analysis, which in the present case resulted in 18–32 % of the total number of features in the authentic data batches being discarded (Suppl. Table 2). Allowing a less restrictive CV limit admits additional noise into the variables and subsequent statistics, but also more variables of potential interest into the statistical analyses. Noisy, uninformative variables could then be excluded from the data set in a later step, using e.g. statistical methods incorporating unbiased variable selection (Hanhineva et al. 2015a; Buck et al. 2016).

In addition to drift correction and alignment, batch data are typically intensity-normalised, although in some cases this may not be required, e.g. when employing fold-changes between matched samples within batch as input data for statistical modelling (Jonsson et al. 2015). Similarly to within-batch intensity drift correction, unbiased fitness estimation of normalisation performance should ideally be carried out, although a thorough review of the literature could not reveal established methods for this practice.

For the authentic data, feature-wise between-batch normalisation using either long-term reference samples (with the caveats described above) or sample population was decided using a heuristic dual criterion quality indicator (Fig. 1c), where the first criterion (Eq. 3) assessed the precision of the reference sample intensity for the specific feature and the second criterion (Eq. 4) was a proxy for assessment of accuracy, under the assumption that large deviations from the general intensity ratio between batches indicates inaccuracy. With increasing limits of intensity ratio allowance, an increasing proportion of features are normalised by reference sample intensities (Suppl. Fig. 6). When long-term references were not considered representative of the sample population, as indicated by the heuristic, batches were instead normalised by sample population median under the assumption of similar batch population distributions. For visualization purposes, between-batch normalization was performed on the actual study data from 562 samples in 3 analytical batches. When observed in a PCA, samples were initially observed to cluster according to batch (Fig. 4d), whereas these systematic differences were removed after normalization (Fig. 4e).

The algorithms developed for within- and between-batch correction are available as an R package (‘*batchCorr’*), which allows ease of implementation. These algorithms can be used either alone or in combination to suit any particular analytical situation. For example, within-batch correction without alignment or normalisation can be applied if samples are analysed within only one batch. Moreover, in the case of multiple batches, these algorithms can easily be chosen at will, combined with other drift correction and/or normalisation procedures and incorporated into a customised workflow. The internal application order of the batch correction algorithms developed also leaves freedom of choice as to whether to perform drift correction or alignment first. In the present study, alignment was performed on an entire dataset containing all features, rather than batch-specific subsets with non-similar features present. Moreover, clustering of variables in within-batch drift correction was improved through the removal of noisy non-relevant features by the alignment procedure.

## Conclusions

An approach including multiple algorithms for within- and between-batch correction was developed to overcome some of the measurement errors in LC-MS metabolomics data and thereby improve the quality of data used for statistical analysis. Alignment of peaks systematically misaligned between batches improved the quality of the dataset by merging features otherwise split between batches. This was achieved by aggregating presence/missingness on batch level and combining similar features orthogonally present between batches. Signal intensity drift correction by clustering of features in the observation space increased within-batch data quality by allowing for multiple drift patterns within the same batch. It also minimised the risk of overfitting (e.g. modelling of noise in individual features) by adding statistical strength of multiple features to the individual cluster regressions. Between-batch correction strategies must correspond to the experimental setup at hand. Long-term reference or QC samples are not necessarily representative of the sample population and normalisation of such features can easily introduce severe batch bias. A heuristic indicator was developed to assess the suitability per feature to utilise different normalisation techniques, i.e. reference-based or population-based between-batch normalisation. Care should be taken to employ unbiased measures for quality improvement using data correction techniques to avoid overfitting and introducing bias.

## Electronic supplementary material

Below is the link to the electronic supplementary material.

Supplementary material 1 (DOCX 591 kb)
