# Supplementary material for: Large-scale untargeted LC-MS metabolomics data correction using between-batch feature alignment and cluster-based within-batch signal intensity drift correction
Source: Metabolomics. 2016 Sep 22;12(11):173. doi: 10.1007/s11306-016-1124-4 (PMC5031781; doi:10.1007/s11306-016-1124-4)
Supplement: Supplementary file 1 — Supplementary material 1 (DOCX 591 kb) [file 11306_2016_1124_MOESM1_ESM.docx]

**Supplementary Table 1**: Synthetic data representing four distinct drift patterns (variables A-D), with quality control samples being injected regularly every 5^th^ sample injection.

| Observation | Injection # | Variable A | Variable B | Variable C | Variable D |
| --- | --- | --- | --- | --- | --- |
| 1 | 1 | 1 | 1 | 1.00 | 4.0 |
| 2 | 6 | 1 | 2 | 0.80 | 3.5 |
| 3 | 11 | 1 | 3 | 0.70 | 3.0 |
| 4 | 16 | 1 | 4 | 0.65 | 3.5 |
| 5 | 21 | 1 | 5 | 0.70 | 3.0 |
| 6 | 26 | 1 | 5 | 0.80 | 2.8 |
| 7 | 31 | 1 | 5 | 1.00 | 2.5 |
| 8 | 36 | 1 | 5 | 1.10 | 2.7 |
| 9 | 41 | 1 | 5 | 1.20 | 3.0 |
| 10 | 46 | 1 | 5 | 1.00 | 3.5 |

**Supplementary Table 2:** Number of features at different batch correction stages: a) between batch alignment (Fig. 1); b) cluster-based within batch intensity drift correction (Fig. 3) and; c) features present in all batches after intensity drift correction (Fig. 5).

| Features  (from XCMS) | After batch  alignment^a^ | After drift  correction^b^ | After combining  batches^c^ |
| --- | --- | --- | --- |
| 11 815 |  | *Batch B*: 9233 | 6025 |
|  | 11 298 | *Batch F*: 7566 |  |
|  |  | *Batch H*: 9146 |  |

**
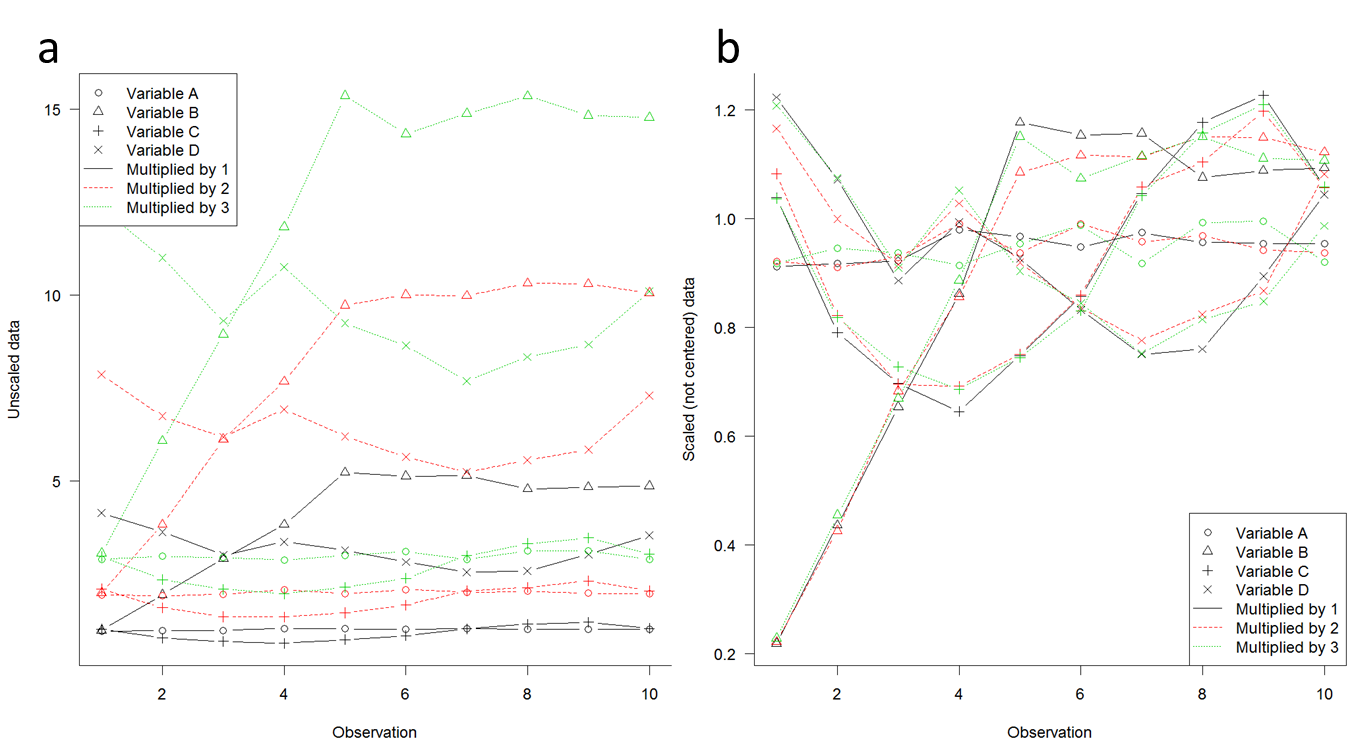
**

**Supplementary Figure 1:** a) Unscaled and b) scaled synthetic data representing four distinct drift patterns (Table 1) at three different levels with ≤5% random noise added to all data points. Note how clusters are visibly discernible among scaled, but not unscaled, variables.


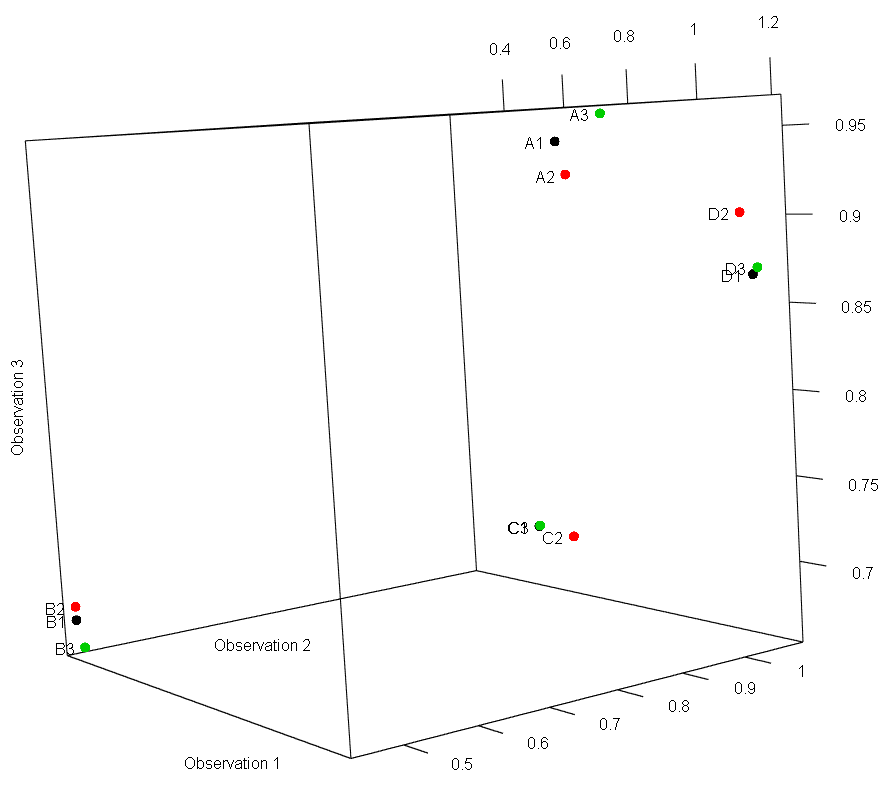


**Supplementary Figure 2:** Twelve scaled synthetic variables as coordinates in observation space (3D representation in the first three observations). Variables are visibly grouped in four distinct clusters corresponding to the original drift types (Supplementary Table 1). Variables are named by original drift type (A-D) and multiplier of original drift variable (1-3).


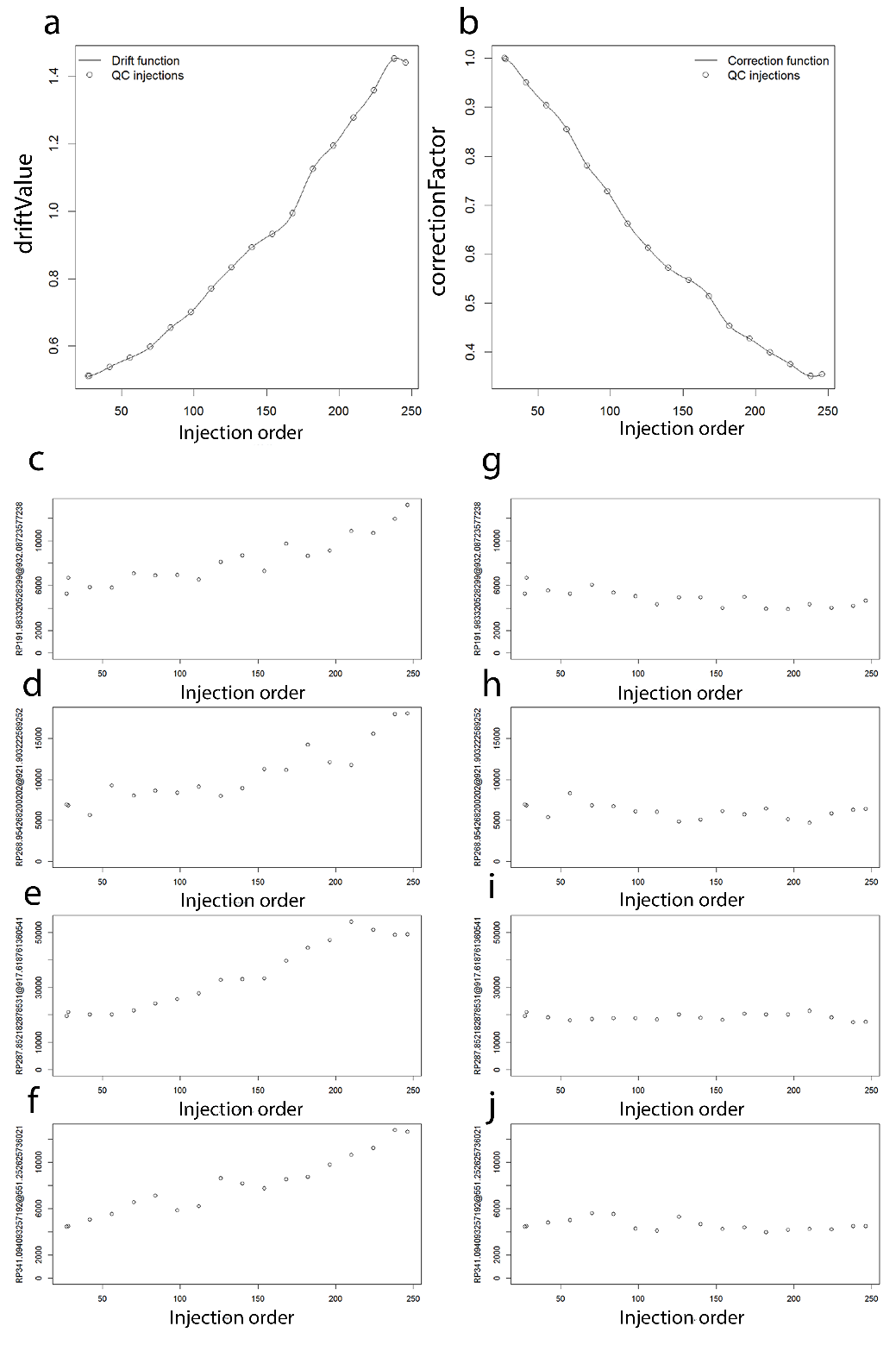


**Supplementary Figure 3**: The performance of cluster-based drift correction on randomly selected individual features within a cluster. The combined cluster QC drift function (**a**) is used to generate corresponding correction factors (**b,** Eq. 2). These correction factors are directly multiplied to the original data within the cluster (**c-f**, randomly selected features), thus generating cluster-based intensity-drift corrected features (**g-j**).

**Supplementary Figure 4**: Strategies to deal with missing data resulting from systematic misalignment across batches exemplified in the retention time domain. Increasing bandwidth setting to capture all batches (black lines) introduces the risk of forcing alignment of unrelated features. Lower bandwidth settings can accurately capture batch-specific features (dashed blue, whole green or dot-dashed red), but may result in missing data after peak picking. Forced filling will insert mostly baseline noise (approx. zero areas) into the peak table, whereas imputation will provide estimates of the true values. Batch alignment will provide the possibility to accurately aggregate batch-specific features to their global counterparts.


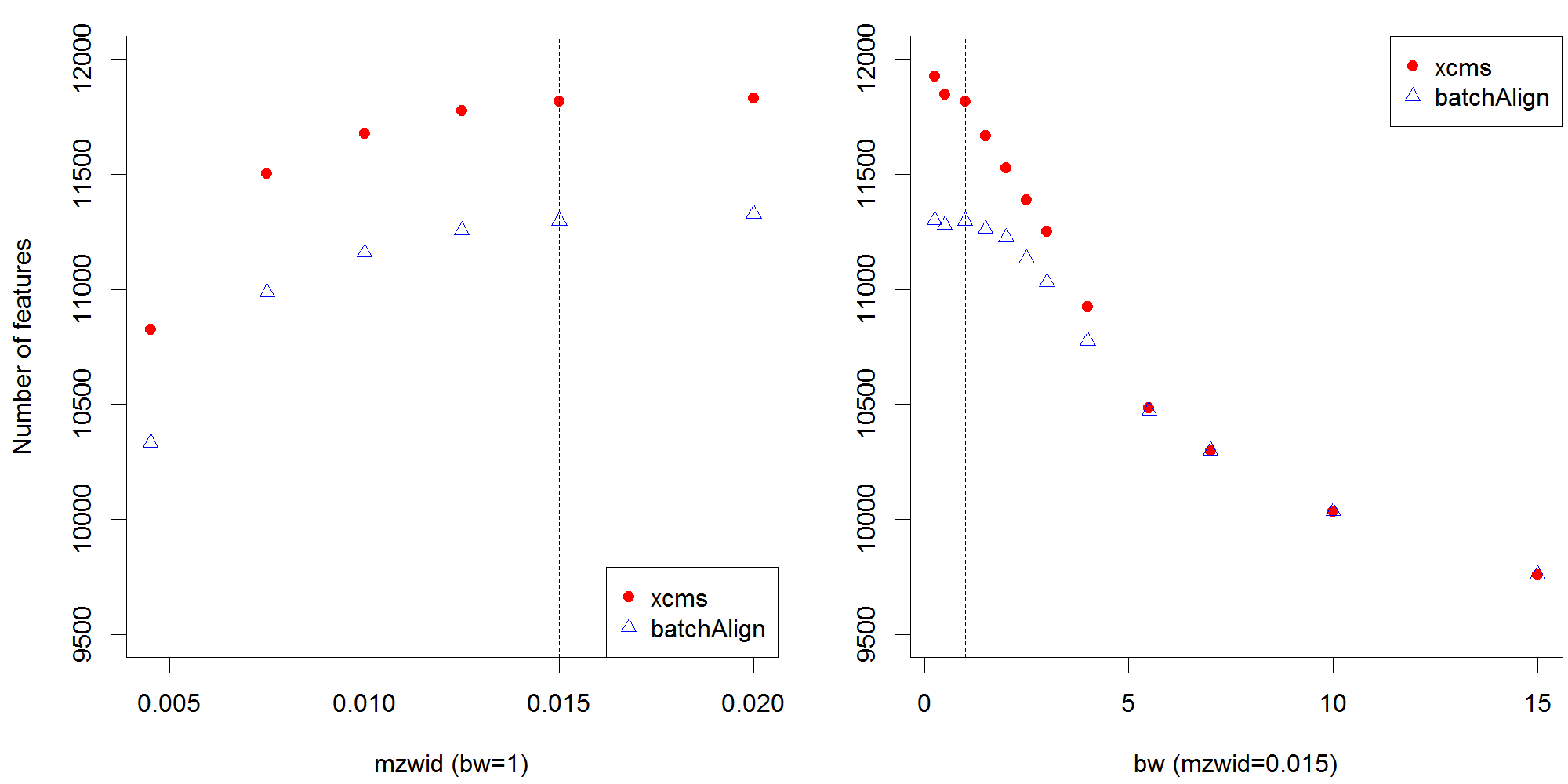


**Supplementary Figure 5**: Number of features present in three authentic data sets as a function of mass accuracy (left) and retention time (right) tolerance settings during alignment. Number of features are reported as naïvely picked by the xcms algorithm (red circles) and after batch alignment of features systematically misaligned between batches (blue triangles). Final xcms alignment parameters (dotted vertical lines) were chosen to optimise the number of reproducibly picked and batch-aligned features. Decreasing retention time bandwidth (bw) setting from 15 s (maximum retention time shift among samples) to 1 s (reproducible batch-specific features) resulted in an increase in number of picked features from 9800 to 11 800, of which 500 were in fact true features artificially split between batches and combined through the batch alignment algorithm. The resulting increase of 1500 features represents an increased information content, but also deconvolution and noise reduction from 1500 of the 9800 features otherwise picked.


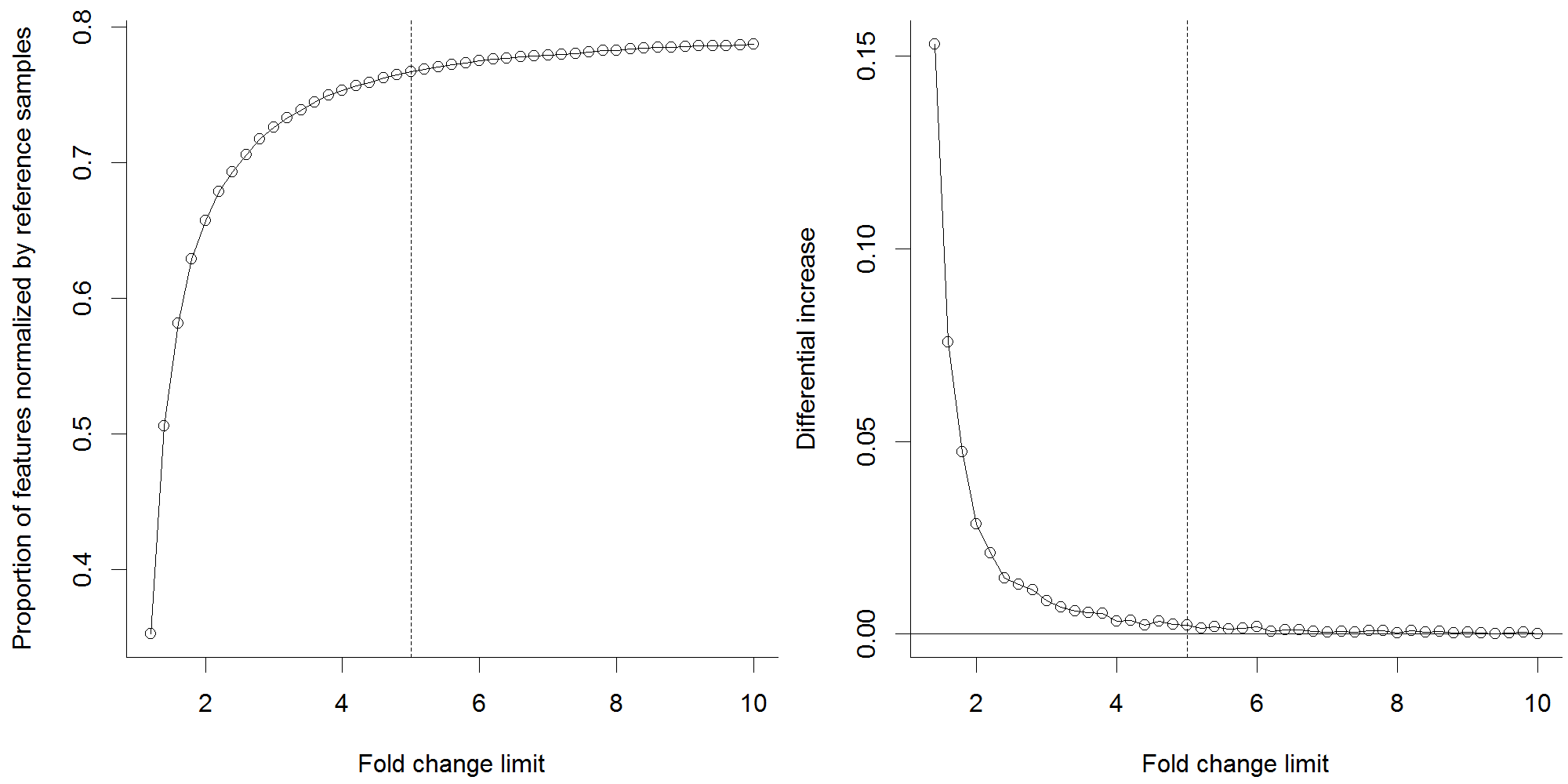


**Supplementary Figure 6**: Proportion (left) and differential increase (right) of features batch-normalised by long-term reference samples as a function of maximum allowance for reference feature intensity difference between batches (Eq. 4). A fold change limit of 5 allowed a majority of features to be normalised by this approach, with close to zero differential increase. Other features were instead normalised by sample population median values.
